# Safeguarding Imperiled Biodiversity and Evolutionary Processes in the Wallacea Center of Endemism

**DOI:** 10.1093/biosci/biac085

**Published:** 2022-10-19

**Authors:** Matthew J Struebig, Sabhrina G Aninta, Maria Beger, Alessia Bani, Henry Barus, Selina Brace, Zoe G Davies, Maarten De Brauwer, Karen Diele, Cilun Djakiman, Rignolda Djamaluddin, Rosie Drinkwater, Alex Dumbrell, Darren Evans, Marco Fusi, Leonel Herrera-Alsina, Djoko T Iskandar, Jamaluddin Jompa, Berry Juliandi, Lesley T Lancaster, Gino Limmon, Lindawati, Michaela G Y Lo, Pungki Lupiyaningdyah, Molly McCannon, Erik Meijaard, Simon L Mitchell, Sonny Mumbunan, Darren O'Connell, Owen G Osborne, Alex S T Papadopulos, Joeni S Rahajoe, Rosaria, Stephen J Rossiter, Rugayah, Himmah Rustiami, Ulrich Salzmann, Sheherazade, I Made Sudiana, Endang Sukara, Johny S Tasirin, Aiyen Tjoa, Justin M J Travis, Liam Trethowan, Agus Trianto, Tim Utteridge, Maria Voigt, Nurul Winarni, Zulianto Zakaria, David P Edwards, Laurent Frantz, Jatna Supriatna

**Affiliations:** Durrell Institute of Conservation and Ecology, University of Kent, Canterbury, England, United Kingdom; School of Biological and Behavioural Sciences, Queen Mary University of London, London, England, United Kingdom; School of Biology, University of Leeds, Leeds, England, United Kingdom; School of Life Sciences, University of Essex, Colchester, England, United Kingdom; Faculty of Agriculture, Tadulako University, Palu, Indonesia; Department of Earth Sciences, Natural History Museum, London, England, United Kingdom; Durrell Institute of Conservation and Ecology, University of Kent, Canterbury, England, United Kingdom; Commonwealth Scientific and Industrial Research Organisation (CSIRO), Hobart, Australia; School of Applied Sciences, Edinburgh Napier University, Edinburgh, Scotland, United Kingdom; Maritime and Marine Science Center of Excellence, Pattimura University, Ambon, Indonesia; Faculty of Fishery and Marine Science, Sam Ratulangi University, Manado, Indonesia; School of Biological and Behavioural Sciences, Queen Mary University of London, London, England, United Kingdom; School of Life Sciences, University of Essex, Colchester, England, United Kingdom; School of Natural and Environmental Sciences, Newcastle University, Newcastle upon Tyne, England, United Kingdom; School of Applied Sciences, Edinburgh Napier University, Edinburgh, Scotland, United Kingdom; School of Biological Sciences, University of Aberdeen, Aberdeen, Scotland, United Kingdom; School of Life Sciences and Technology, Bandung Institute of Technology, Bandung, Indonesia; Faculty of Marine Science and Fisheries, Hasanuddin University, Makassar, Indonesia; Department of Biology, Bogor Agricultural Institute, Bogor, Indonesia; School of Biological Sciences, University of Aberdeen, Aberdeen, Scotland, United Kingdom; Department of Marine Science, Pattimura University, Ambon, Indonesia; Department of Chemical and Green Process Engineering, Surya University, Tangerang, Indonesia; Durrell Institute of Conservation and Ecology, University of Kent, Canterbury, England, United Kingdom; Research Center for Biosystematics and Evolution, National Research and Innovation Agency, Cibinong, Indonesia; School of Biology, University of Leeds, Leeds, England, United Kingdom; Borneo Futures, Bandar Seri Begawan, Brunei Darussalam; Durrell Institute of Conservation and Ecology, University of Kent, Canterbury, England, United Kingdom; Research Center for Climate Change, University of Indonesia, Depok, Indonesia; School of Biology and Environmental Science, University College Dublin, Dublin, Ireland; Molecular Ecology and Evolution Bangor, Bangor University, Bangor, Wales, United Kingdom; Molecular Ecology and Evolution Bangor, Bangor University, Bangor, Wales, United Kingdom; Research Center for Ecology and Ethnobiology, National Research and Innovation Agency, Cibinong, Indonesia; Department of Biology, University of Oxford, Oxford, England, United Kingdom; School of Biological and Behavioural Sciences, Queen Mary University of London, London, England, United Kingdom; Herbarium Bogoriense, National Research and Innovation Agency, Cibinong, Indonesia; Herbarium Bogoriense, National Research and Innovation Agency, Cibinong, Indonesia; Department of Geography and Environmental Sciences, Northumbria University, Newcastle upon Tyne, England, United Kingdom; Sulawesi Regional Ecological Conservation Initiative, Luwuk, Indonesia; Research Center for Applied Microbiology, National Research and Innovation Agency, Cibinong, Indonesia; Graduate School, University of Indonesia, Depok, Indonesia; Department of Forest Sciences, Universitas Sam Ratulangi, Manado, Indonesia; Faculty of Agriculture of Tadulako University, Palu, Indonesia; School of Biological Sciences, University of Aberdeen, Aberdeen, Scotland, United Kingdom; Herbarium Kew, Royal Botanic Gardens Kew, London, England, United Kingdom; Faculty of Fisheries and Marine Science, Universitas Diponegoro, Indonesia; Herbarium Kew, Royal Botanic Gardens Kew, London, England, United Kingdom; Durrell Institute of Conservation and Ecology, University of Kent, Canterbury, England, United Kingdom; Research Center for Climate Change, University of Indonesia, Depok, Indonesia; Department of Biology, Gorontalo University, Gorontalo City, Indonesia; School of Biosciences, University of Sheffield, Sheffield, England, United Kingdom; School of Biological and Behavioural Sciences, Queen Mary University of London, London, England, United Kingdom; Research Center for Climate Change, University of Indonesia, Depok, Indonesia

**Keywords:** conservation, evolution, interdisciplinary science, tropical ecosystems, applied ecology

## Abstract

Wallacea—the meeting point between the Asian and Australian fauna—is one of the world's largest centers of endemism. Twenty-three million years of complex geological history have given rise to a living laboratory for the study of evolution and biodiversity, highly vulnerable to anthropogenic pressures. In the present article, we review the historic and contemporary processes shaping Wallacea's biodiversity and explore ways to conserve its unique ecosystems. Although remoteness has spared many Wallacean islands from the severe overexploitation that characterizes many tropical regions, industrial-scale expansion of agriculture, mining, aquaculture and fisheries is damaging terrestrial and aquatic ecosystems, denuding endemics from communities, and threatening a long-term legacy of impoverished human populations. An impending biodiversity catastrophe demands collaborative actions to improve community-based management, minimize environmental impacts, monitor threatened species, and reduce wildlife trade. Securing a positive future for Wallacea's imperiled ecosystems requires a fundamental shift away from managing marine and terrestrial realms independently.

Islands offer fascinating insights into the world's   evolutionary processes. Perhaps nowhere is this more evident than the archipelagos of Wallacea, where the Asian and Australasian biogeographic regions collide. Wallacea, which is made up of the islands of Sulawesi, the Moluccas, and Lesser Sunda in Indonesia and Timor Leste, supports the highest levels of endemism worldwide (Mittermeier et al. 2011), including 62 endemic vertebrate genera and iconic oddities such as the babirusa (*Babyrousa* spp.), the maleo (*Macrocephalon* spp.), and the Komodo dragon (*Varanus komodoensis*). Recognizing that the fauna was so strikingly different from that of neighboring Borneo and Papua, nineteenth century naturalist Alfred Russel Wallace changed the course of evolutionary theory by delineating what ultimately became a distinct biogeographic region (figure 1). Although the bounds of Wallacea have, at times, included other islands, it is the central transition zone between the Sahul and Sunda land masses in Indonesia that has long captured scientific attention (Lohman et al. 2011, Ali and Heaney 2021).

**Figure 1.**
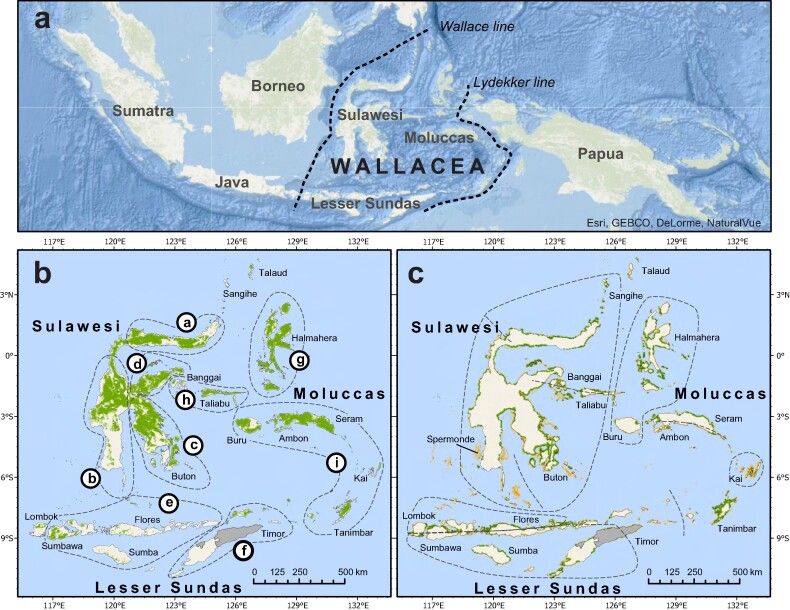
Maps of Wallacea showing its location in Southeast Asia (a), zones of endemism on land (b) and in coastal marine environments (c). See supplemental tables S1 and S2 for further information on the zones. In panel (b), the shading represents Indonesia's primary forest cover in 2018 compiled from Margono and colleagues (2014) and data from www.globalforestwatch.org. Forest according to this definition cannot be reproduced consistently for Timor-Leste, so forest is not displayed for this country. Forests are also difficult to map consistently in the drier parts of Wallacea, notably the Lesser Sunda islands (Nusa Tenggara); see box 1. In panel (c) mangrove cover (green/dark shading) is derived from Bunting and colleagues (2018) and corals (orange/light shading) from https://allencoralatlas.org.

With new marine and terrestrial species discoveries still occurring across the region, the importance of Wallacea as a treasure trove of biodiversity is increasingly evident (Rheindt et al. 2020, Esselstyn et al. 2021). Nonetheless, Wallacea's ecosystems face considerable pressure from a growing human population (33.7 million people in 2021; www.bps.go.id). Economic development and people's livelihoods have largely centered on the exploitation of the region's natural resources, but the consequences of mining, forestry, agriculture, hunting, tourism, and fisheries for biodiversity and particularly for the endemic species that make Wallacea so unique are concerning. In the present article, we review the historic and contemporary processes shaping Wallacea's unique ecosystems and explore ways to conserve its hyperdiversity.

## Wallacea: A natural laboratory for the study of evolution

The abrupt biogeographic transitions of the Wallacean fauna, as is demarcated by Wallace's line to the west and Lydekker's line to the east, are the result of long-term isolation of the archipelago from Asia and Australasia, respectively (Lohman et al. 2011, Ali and Heaney 2021). Faunal migration and speciation were shaped by a complex geological history, involving rifting of the Makassar Strait during the Eocene, collisions of Sunda margins with the Australian continent during the Miocene, and tectonic movement during the early Neogene (Hall 2013). Although most of the small islands in the southeast, as well as Timor and Seram, emerged around 5 million years ago, the largest island, Sulawesi, originally formed as a series of smaller land masses that only amalgamated into its current form around 1 million years ago (Vaillant et al. 2011, Hall 2013, Nugraha and Hall 2018). This merger created distinct volcanic ridges, mountains, and ancient lakes that characterize the island today. Although the formation of Sulawesi as a single island is relatively recent, deep ocean trenches to the east and west have separated it, as well as the wider Wallacea region, from the Sunda and Sahul continental shelves throughout the past 23 million years (Hall 2013).

**Figure 2.**
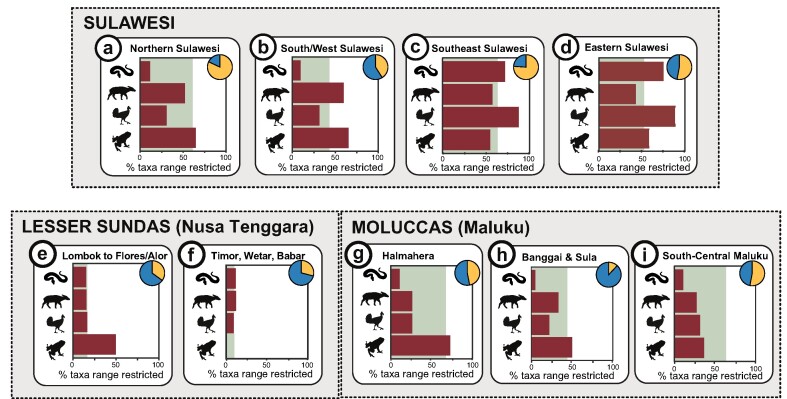
Patterns of terrestrial endemism—as inferred by range restricted reptile, mammal, bird, and amphibian species in the IUCN database—for the eight zones presented in figure 1. Zones are outlined by Michaux (2010) and expanded to include other areas of Sulawesi (figure 1; table S1). Each plot shows the proportion of terrestrial vertebrate species that are range restricted according to www.iucnredlist.org. The assessment is limited to those taxa formally recognized as species and so may underrepresent endemism within species complexes, particularly in parts of Sulawesi (figure 3) and along the island chains of Nusa Tenggara. The shaded background to each plot denotes the percentage of land forested in 2019, and pie charts indicate progress made in each region (percentage in orange or light shade) to meet the country's target of protecting 17% of land by 2020 (based on IUCN categories I–IV for protected areas from www.protectedplanet.net).

This complex geological history and subsequent diverse environments makes Wallacea a natural laboratory for testing the roles of dispersal, geographic isolation, and adaptation in driving evolution, speciation, and ecological diversification. The evolutionary history of the many endemic species that are found in the region's terrestrial and freshwater ecosystems (figure 2) illustrate well how the rise and fall of geological barriers can promote the evolution of species (supplemental tables S1 and S2). The ancestors of many endemic lineages arrived long after the region became isolated from Asia and Australia in the Eocene (Stelbrink et al. 2012), indicating that migration through rafting and island hopping likely played a strong role in the assembly of Wallacea's unique terrestrial ecosystems. There are now more than 120 endemic fishes (e.g., Telmatherininae, sail-fin silversides) and invertebrates (e.g., *Tylomelania* snails and *Syntripsa* crabs) that have subsequently diversified in Sulawesi's ancient lakes, and colonization and isolation in montane regions have triggered further speciation in mammals (*Crocidura*), frogs (*Limnonectes*), and plants (*Cyrtandra*; table S2). Although Wallacea covers less than 2% of the world's oceans, its marine ecosystems contain approximately 76% of the world's hard coral species and more than 2000 shallow noncryptic coral reef fishes (Veron et al. 2009).

**Figure 3.**
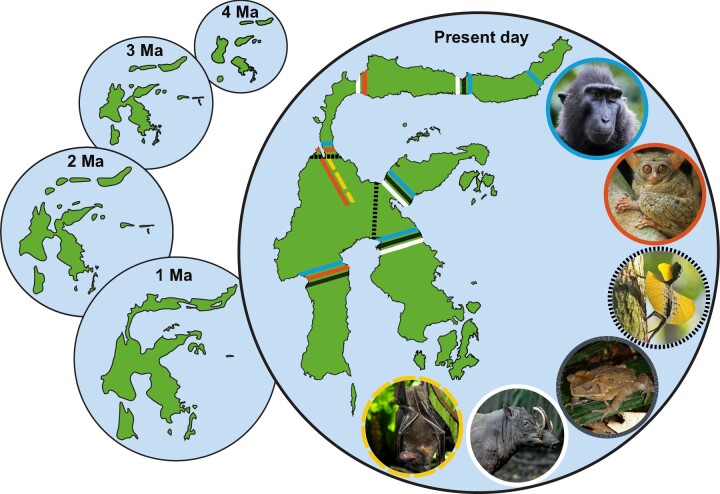
The origins of Sulawesi and some of the resulting contact zones among endemic species or populations in Sulawesi as determined using genetic data. Examples of separated taxa include macaque (Macaca spp.), tarsier (Tarsius spp.), and gliding lizard (Draco spp.). Divergent subpopulations include the Celebes toad (Ingerophrynus celebensis), babirusa (Babyroussa celebensis), and swift fruit bat (Thoopterus nigrescens). Schematic based on Nugraha and Hall (2018). Photographs: Sulawesi babirusa from Simon Mitchell; swift fruit bat Klaus Rudloff; other images from Wikimedia commons.

In some taxa, populations and species likely diversified in isolation on separate paleoislands, perhaps with occasional dispersal across the paleoarchipelago. Subsequent periods of large-scale uplift over the past 4 million years (Nugraha and Hall 2018) had further impacts on biodiversity by connecting previously separated regions (Frantz et al. 2018). Indeed, this uplift may have reunited previously isolated species and subspecies of terrestrial taxa, including tarsiers, shrews, macaques, snails, frogs, lizards, crickets, and damselflies (supplemental table S3), generating distinctive phylogeographic patterns (figure 3) and some generalized zones of terrestrial endemism (figures 1 and 2, table S1; Michaux 2010). However, there are many lineages that buck these trends. For example, many freshwater lineages (e.g., *Tylomelania* snails *Caridina* shrimps, Gecarcinucid crabs and Telmatherinid fish) have undergone adaptive radiations in the island's ancient lake systems (von Rintelen et al. 2012), and plant diversification (e.g., *Cyrtandra* and *Nepenthes*) may be more heavily influenced by soil types, elevation, and other ecological barriers (Bouchenak-Khelladi et al. 2015).

Wallacea's complex geological history, combined with its location and the extent of its coastline at the confluence of the Pacific and Indian Oceans, also promoted the evolution of exceptionally biodiverse coastal marine ecosystems (Tittensor et al. 2010, Wicaksono et al. 2017). The archipelagos of the region lie in the center of the Coral Triangle, the global epicenter for marine diversity, including corals, fishes, and mollusks (Veron et al. 2009). Different biogeographical hypotheses have been proposed for how this high biodiversity came to be (Veron et al. 2009, Huang et al. 2018). The region's unstable tectonic environment continually creates diverse shallow habitats. During past sea level changes, these often remained connected to deep water, creating refuges that formed both a buffer from extinction and opportunities for divergent evolution of endemic species. The consistent movement of water and eddies created by the Indonesian throughflow ocean current increases the chance of subsequent larval dispersion at different rates across geological timescales (e.g., Linsley et al. 2010).

Wallacea's paleontological record bears witness to these complex evolutionary processes and to the emergence of large numbers of island endemics (figure 2). Although less is known about Wallacea's fossil record compared with that of neighboring Java, the oldest terrestrial fauna recorded (approximately 2.5 million years ago) from South Sulawesi includes giant suids (*Celebochoerus*), giant tortoises (*Geochelone atlas*), and two dwarf elephants (*Elephas celebensis* and *Stegodon sompoensis*; van den Bergh et al. 2001). Most of these megafauna became extinct at the end of the Pleistocene or the early Holocene. The paleontological record is also rich in hominid (other than *Homo sapiens*) fossils and artifacts, which first appeared in the Pleistocene around 1 million years ago (van den Bergh et al. 2001). Given that the region was never connected via a land bridge to neighboring continental shelves, these hominids would have had to complete sea crossings across treacherous waters to reach Wallacean islands with limited technologies, most likely from Borneo (Shipton et al. 2020). Wallacea was likely inhabited by at least two early hominid species, including *Homo floresiensis* and, potentially, *Homo erectus* (van den Bergh et al. 2001), both of which disappeared by the end of the Pleistocene, together with other megafauna (Sutikna et al. 2018). Archeological evidence for the presence of modern humans (*Homo sapiens*) indicated that they may have lived on Wallacean islands around 50,000–30,000 years ago (Shipton et al. 2020), with evidence of their activities including some of the earliest prehistoric cave paintings dated to at least 40,000 years ago (Brumm et al. 2021).

The activities of hominins, including modern humans, likely had a strong impact on the region for thousands of years. Even prior to the introduction of farming, at least 40 taxa were thought to have been translocated across Southeast Asia to Wallacean islands, including game species such as deer, pig, buffalo, and junglefowl (Heinsohn 2003). These translocations likely increased with the arrival of modern humans in the archipelago (Kealy et al. 2017), probably for hunting stock or ceremonial purposes. The process further intensified with the introduction of domesticated taxa, including pigs, dogs, and chickens, which then became feral during the expansion of farming cultures from mainland Asia (Piper 2017).

## Contemporary human pressures

**Figure 4.**
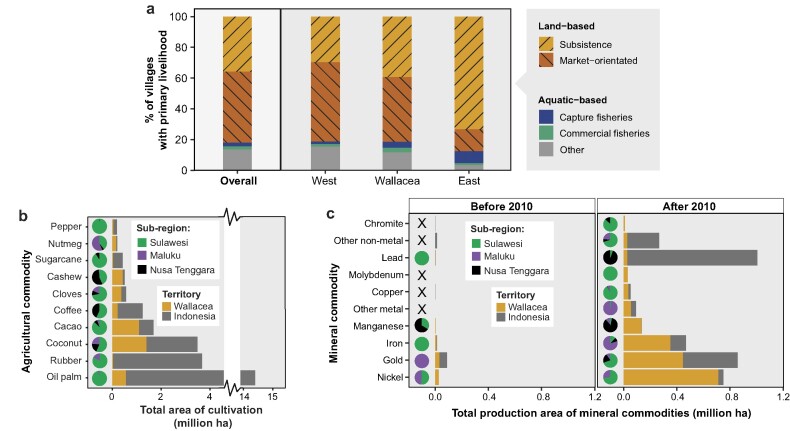
Livelihood systems, cultivated crops and mining commodities in Wallacea compared with other parts of Indonesia. In panel (a), the primary livelihoods at the village level are shown according to Indonesia's Village Potential census in 2018. Subsistence livelihoods include subsistence agriculture, swidden agriculture, hunting and gathering of forest products and capture fisheries; market-orientated livelihoods include monoculture, horticulture, logging, animal breeding, aquaculture, and commercial fisheries. Western Indonesia includes Java, Sumatra, and Kalimantan; Wallacea  is Sulawesi, Maluku and Nusa Tenggara; Eastern Indonesia includes Papua, West Papua and nearby islands. Panel (b) shows the total cultivated area of the 10 dominant agricultural commodities in Wallacea and Indonesia more widely, according to the Directorate General of Estate Crops Indonesia in 2019. Panel (c) shows the total concession area under production for the 10 most dominant mineral commodities in Wallacea a decade before and after mining policy changes  in 2010, according to the Ministry of Energy and Mineral Resources.

**Figure 5.**
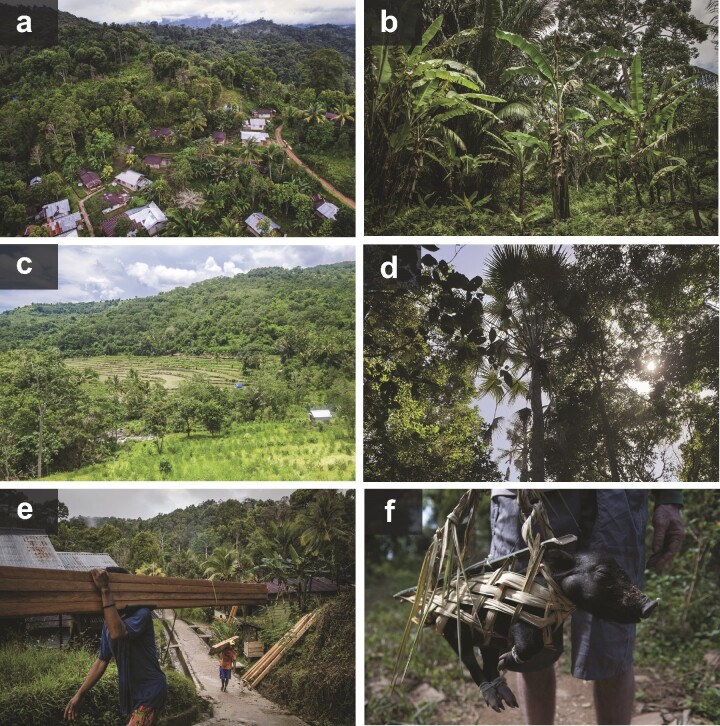
Forest and forest uses in Wallacea. (a, b) Agroforestry practiced in community managed forest of Seram, Maluku raises the question of how to define a forest from aerial imagery. (c, d) Seasonal forests in Sumbawa, Nusa Tenggara are mostly deciduous and fragmented by small-scale agriculture. (e, f) Examples of the many uses of forests for local communities include timber extraction (in the present figure, Seram, Maluku) and subsistence hunting (Flores, Nusa Tenggara). Photographs: Aris Santaya (a), Ulat Ifansasti (b, e) and Aulia Erlangga (c, f), courtesy of CIFOR.org, and Gemma Bramley (d), Royal Botanic Gardens Kew.

More than 160 years after Wallace wrote of the rich marine diversity of Ambon, the coast of this Moluccan island has been dredged, overfished, and polluted (Limmon and Marasabessy 2019). This provides just one example of the multiple stressors that have affected Wallacean environments in recent decades. But the remoteness of Wallacea, especially its eastern islands, has spared much of the region from the severe overexploitation of marine, freshwater, and terrestrial ecosystems characteristic of other parts of Southeast Asia. For instance, the rugged interior forests of most Wallacean islands remain relatively intact (Voigt et al. 2021), having avoided the pervasive conversion to large-scale plantations typical of western Indonesia, Malaysia, and southern Thailand. Indeed, between 2001 and 2019 the annual deforestation rate across Wallacea (0.39%) was half of that of neighboring Borneo (0.76%) and a quarter of that of Sumatra (1.52%; figure 1; Gaveau et al. 2022), reflecting the continued dominance of small-scale farming and agroforestry over large-scale extractive industries (figures 4 and 5). Compared with industrial agriculture, these low-intensity farmlands support high species diversity, especially when close to forest and where habitat heterogeneity and canopy cover is maximized (Waltert et al. 2011). Nonetheless, forest specialists and endemic species are disappearing from these communities, often being replaced by wide-ranging generalists following a process of biotic homogenization across the archipelago (Maas et al. 2009, Mitchell et al. 2022).

A deforestation surge over the last 10 years, primarily in Central Sulawesi and northern Maluku, has been linked to the expansion of mining and industrial oil palm plantations (Supriatna et al. 2020). Although the region's oil palm industry is at an early stage, mineral extraction has rapidly expanded since 2010 following a new mining governance regime in Indonesia. This led to more than 95% of the country's nickel coming from Wallacean islands (mainly Sulawesi and Halmahera), and around half of its gold (figure 4). Elsewhere, key agricultural commodities, such as coconut and cacao (figure 4), have resulted in small-scale but widespread encroachment of forest areas (Supriatna et al. 2020), whereas mangroves have been replaced by commercial shrimp farms (Richards and Friess 2016, Malik et al. 2017).

Land-cover changes also leave Wallacea's islands particularly susceptible to ecological invasion. Human-mediated establishment of invasive species can occur by either facilitating dispersal across islands after natural arrival or introducing those species from elsewhere. For example, genetic analyses suggest seven amphibian and reptile taxa that are now widespread in human settlements and croplands of the Lesser Sundas colonized these islands relatively recently (less than 100 years ago; Reilly et al. 2019). Invasion of the poisonous Asian common toad (*Duttaphrynus melanostictus*) is a key concern because the species has benefited from the absence of toads in the region and expanded quickly throughout. The large snakes and varanid lizards at the top of Wallacea's food chains are particularly susceptible to poisoning from eating this prey, because, as naive predators, they are incapable of processing the toad's toxins (Reilly et al. 2017). Sulawesi's lake ecosystems have also been adversely affected by introduced species, including flowerhorn cichlids, Nile tilapia, and common carp (Serdiati et al. 2021). For example, in Matano, one of South Sulawesi's ancient lakes, adult flowerhorn cichlids prey on native fish and invertebrates endemic to this ancient lake (Hilgers et al. 2018). In Central Sulawesi's Lake Poso, this predation and competition is so severe that the number of nonnative fish species (17) now exceeds the native fish fauna (13; Herder et al. 2022).

The infrastructure development that often accompanies deforestation has facilitated hunting and wildlife trade throughout Wallacea, which is challenging to counter in more remote islands, where patrols, communication, and enforcement are limited. Wild meat is routinely sold in markets and supermarkets in North Sulawesi, for example, and around 500 metric tons of bats are imported to the province annually from across the island (Sheherazade and Tsang 2015). Egg collection of Sulawesi's famed maleo birds (*Macrocephalon maleo*) has led to a rapid abandonment of nesting grounds and the species elevated to Critically Endangered status by the IUCN (www.iucnredlist.org/species/22678576/194673255). In Maluku, trade in endemic birds for the pet and songbird trade is prolific, having caused an 80% crash in salmon-crested cockatoo (*Cacatua moluccensis*) populations in a decade (www.iucnredlist.org/species/22684784/93046425). Because Wallacea is characterized by relatively few large-body terrestrial vertebrates, unsustainable extraction levels disproportionately affect a small set of these highly threatened species (Scheffers et al. 2019).

Landscape change and overexploitation have important ramifications for Wallacea's aquatic ecosystems. Hydroelectric dams and mining pose huge threats to the integrity and functioning of freshwaters, culminating in Sulawesi ancient lakes (von Rintelen et al. 2012). A gold rush in North Sulawesi, for example, led to elevated mercury concentrations in nearby rivers and high bioaccumulation in fish (Limbong et al. 2003). Runoff from agricultural practices and urbanization has reduced downstream water quality in Ambon Bay to the extent that the sediment load smothers corals and has shifted reef communities toward sediment-tolerant species (Limmon and Marasabessy 2019). Pollution of the marine environment tends to be stronger close to major population centers, such as Makassar and Manado in Sulawesi, which also attract substantial fishing pressure. In contrast, the eastern islands of Maluku are less exposed to commercial fisheries, but artisanal fishing is widespread, and its impacts are poorly understood. Many of these islands still host the highest reef fish biomass in Indonesia (Campbell et al. 2020). However, broadscale, destructive fishing practices are evident even in remote reefs, leaving a long-term legacy of damaged reef structure and overexploited populations (e.g., overfishing of endangered Banggai cardinalfish, *Pterapogon kauderni*, for the aquarium trade, www.iucnredlist.org/species/63572/12692964).

Climate change further threatens Wallacean biodiversity, with at least two recent coral bleaching and mortality events degrading coral habitats, fisheries, and, therefore, income opportunities (Moore et al. 2017). The reefs around Buton and Spermonde islands off Sulawesi, for instance, show a community shift from a dominance of branching corals (e.g., *Acropora* spp.) toward more heat-tolerant species (Yusuf and Jompa 2012). Climate change also pushes terrestrial species outside of their thermal optima, changing phenology, and seasonality that could drive transitions toward savanna (Siyum 2020), making dry forest ecosystems of the Lesser Sunda islands (e.g., Sumbawa, Flores; box 1) particularly vulnerable. In the absence of adaptation or behavioral and phenological changes, species will have to move to track their thermal envelopes, and much of that movement on Wallacea's rugged and convoluted islands is expected to be upslope (Harris et al. 2014). Although this high landscape complexity provides potential refuge areas that may help buffer some species against the most adverse impacts of climate change (Trew and Maclean 2021), the high numbers of species endemic to single islands or small island chains mean that the potential for mountaintop extinctions is high should these refuges prove insufficient. This is particularly concerning for species with limited dispersal capabilities or those highly dependent on intact habitat or water resources on drier and more seasonal islands in the east. These issues point toward the need for integrated land and coastal management to retain connectivity of protected forests, mangroves, and coral reefs across thermal gradients.

**Box 1. Characterizing the forests of Wallacea.**

Mapping and monitoring forests is essential for countries to meet their sustainability commitments, such as conserving biodiversity and ecosystem processes and tackling climate change (e.g., Nadin et al. 2019). But monitoring the fate of Wallacea's forests is surprisingly difficult, even with advances in remote sensing. In 2018, the islands of Sulawesi, Maluku, and Nusa Tenggara represented 141,080 square kilometers (km^2^) of forest according to Global Forest Change data, a reduction of 10,231 km^2^ since 2000 (Voigt et al. 2021). However, like any mapping assessment (e.g., Austin et al. 2019), this coverage remains prone to error because of ongoing challenges in distinguishing forest from regrowth and forest-like vegetation, particularly agroforestry (e.g., Sulawesi, Halmahera, and Seram; figure 5a, b). This is compounded in the islands of southern Maluku and Nusa Tenggara where the tropical moist forests characteristic of equatorial Southeast Asia become much drier seasonal ecosystems (figure 5c). Because seasonal forests have different spectral properties to tropical forests, their extent is likely underestimated in large-scale mapping assessments that rely solely on standard forest definitions (Staver et al. 2011).

**Figure 6.**
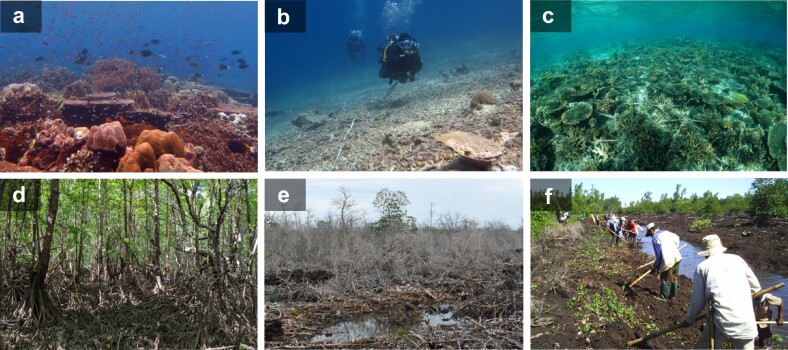
Intact (a) and degraded (b) coral reef in North Sulawesi, and 2 years of restoration in Spermonde, South Sulawesi (c). Old growth (d) and degraded (e) mangrove forests in North Sulawesi, followed by community-based hydrological restoration (f)—that is, a channel that was reopened to connect the old shrimp pond area to the sea. Photographs: Maarten De Brauwer (a, b), Dominic Muenzel (c), Karen Diele (d, e), Rignolda Djamaluddin (f).

The islands of the Lesser Sundas (i.e., Nusa Tenggara) lie within a climatic zone that could support both dry forest and savanna (Staver et al. 2011). The flora are tolerant to drought and the fauna are somewhat adapted to the phenology of these predominantly deciduous forests. Unlike savanna, seasonal forests of Nusa Tenggara include a closed canopy and no grass layer (figure 6d; Pennington et al. 2018). Baseline taxonomic knowledge of Wallacea's seasonal forests lags far behind similar ecosystems in the American tropics, where plant diversity is known to rival that of Amazonia's prominent wet tropical forests (Pennington et al. 2018). Current estimates suggest around 300 plant species endemic to the Lesser Sundas can be found in Nusa Tenggara's seasonal forest, but this list continues to grow (e.g., Sunarti et al. 2022).

Key threats to Wallacea's seasonal forests include mining and encroachment from small-scale agriculture (Austin et al. 2019, Voigt et al. 2021), as well as highly localized collection of firewood, timber, plants and wildlife (figure 5e, f). Although in tropical Southeast Asia agricultural plantings tend to be long-term cash crops, the low rainfall of the Lesser Sunda islands limits irrigation. Seasonal forests therefore tend to be replaced by rotating cultivation systems, which, over time, may return to forest vegetation (Monk et al. 1997). Unfortunately, the dry local climate makes seasonal forests highly vulnerable to these activities. A single stressor—such as fire used to clear land—can rapidly alter the stable state of forest to that of savanna. Although both savanna and seasonal forest plants can cope with drought, differences in fire regimes between these two ecosystems mean they support fundamentally different species (Pellegrini et al. 2021). Seasonal forest plants tend to be highly intolerant to fire, meaning that any switches to savanna are less reversible, posing a great risk in the highly threatened seasonal forests of Nusa Tenggara.

## Wallacea at a crossroads

Sustainable use of natural resources is needed to ensure biodiversity, evolutionary processes, and ecosystem functioning are maintained while accounting for rapidly growing human pressures and accelerating climate change. The situation is further exacerbated in Wallacea by incentivizing development policies that encourage unsustainable practices in agriculture, mining, and fisheries, and therefore, the continued overexploitation of terrestrial and marine ecosystems. On one hand, low-intensity cultivation tends to have fewer major impacts on the local environment relative to large-scale agriculture (Waltert et al. 2011), and deforestation has been much slower in Wallacea than in other regions as a result (Voigt et al. 2021). On the other hand, the income potential of these livelihoods is often insufficient, making some parts of Wallacea (e.g., Maluku, Gorontalo in Sulawesi, and East Nusa Tenggara) among the poorest in Indonesia, whereas those that are better oriented to market-based livelihoods (e.g., North and South Sulawesi) prosper (www.bps.go.id; figure 4). Forested landscapes are often poorly suited to farming, with limited transportation and infrastructure networks, leaving residents facing high costs to access markets, education, and healthcare (Angelsen et al. 2014). Coastal communities face similar challenges with accessing markets and social facilities, particularly on isolated islands. These considerations, combined with ­challenging law enforcement, mean that solutions to Wallacea's emerging biodiversity crisis are not as simple as lobbying for further habitat protection.

Currently, only 9% of Wallacea's land and 3% of its marine zone are formally protected for biodiversity conservation (figure 1; www.protectedplanet.net/country/IDN), although Indonesia has ambitious targets for protected area expansion by 2030. Similarly, although formally protected terrestrial reserves have curbed deforestation in endemic-rich areas (Voigt et al. 2021), they are limited in Buru and Flores, and many of the smaller islands. Protected areas, particularly marine ones, face many logistic, managerial, and funding challenges to be effective (Kamil et al. 2017). Moreover, expanding strictly protected areas places substantial burdens on natural resource users and are often viewed as threatening livelihoods, despite, for instance, the need for no-take zones to support sustainability of fisheries (Mills et al. 2010). It is therefore imperative to work with local communities when establishing protected areas or other conservation interventions. Much of the current conservation activity in Wallacea is focused on protected areas and where international nongovernmental organizations operate. Diversifying conservation resources, as well as increasing them, will help empower grassroots organizations and multiple stakeholders so that conservation could be more locally led.

Community-based management has a long history on land and at sea and, in Indonesia, is manifested as highly localized customary *adat* practices. Since 2015, these customary rights have been embodied within Indonesia's social forestry program and extended to other local communities, regardless of cultural or ethnic background. Among the multiple social forestry schemes available, those that permit some limited timber extraction and agroforestry for local use can help improve social welfare while minimizing deforestation (Rakatama and Pandit 2020). However, uptake across the Wallacean region has been slow relative to the western islands of Indonesia (Santika et al. 2019). Correspondingly, marine community-based management (e.g., Territorial Use Rights in Fisheries) typically allows some resource extraction and can achieve sustainable fish biomass. Culturally embedded conservation practices of the sea or land in Maluku pertain to local dynamic closures that prohibit and allow fishing. Contextualizing these practices within traditional marine tenure and belief systems—*sasi*—has been effective in reducing overfishing (Halim et al. 2020).

Community involvement and local leadership are also vital for restoring ecosystem function and diversity. Some of the best examples come from the coastal–marine zone, where communities have been instrumental in restoring reefs and mangroves, albeit after many failed attempts (box 2). On land, Indonesia also pioneered the use of ecosystem restoration licenses through which degraded forests are leased by the government for restoration-compatible business development (Harrison et al. 2020). Licenses are granted typically for 60–100 years, allowing activities such as conservation, ecotourism, management of nontimber forest products, and tree protection for carbon sequestration. Once legally harvestable timber volumes have been restored and environmental safeguards met, a license holder may log the forest. Around one-third of the forest eligible for ecosystem restoration licenses (4,325,649 hectares by 2020. htp://phl.menlhk.go.id/tabular) are in the Wallacea region, with most of these located in Sulawesi and Maluku.

**Box 2. Restoring Wallacea's blue ecosystems.**

Wallacea has among the highest mangrove, seagrass and coral biodiversity and biomass globally (Veron et al. 2009, Alongi et al. 2016), providing extensive ecosystem services. But these “blue” ecosystems face unprecedented pressures as aquaculture and farmland replace mangroves, disruptive fishing techniques and tourism affect seagrass meadows and reefs, and ocean warming kills corals. These pressures have affected commercially important fisheries, increased erosion, decreased coastal stabilization, and reduced water quality (Camp et al. 2016), substantially degrading valuable ecosystem services.

Mangrove restoration programs involving monospecific plantations often fail because of a poor choice of foundation species or lack of hydrological restoration. However, in Wallacea, the government, businesses, nongovernmental organizations, and local communities have had some success in restoring blue ecosystems. For example, in Bunaken National Park, local communities broke down the walls of abandoned shrimp ponds and dug trenches to reestablish tidal flow, supporting natural recruitment of mangrove propagules and associated organisms (figure 6; Djamaluddin et al. 2019). The resulting mixed-species regeneration, facilitated by the hydrological interventions (i.e., “ecological restoration”) supported diverse faunal communities and interaction networks that more closely resembled old-growth areas than monoculture plantings of similar age (O'Connell et al. 2022).

There are similar coral reef restoration success stories for the region. Novel community-based reef restoration efforts in Spermonde, South Sulawesi, resulted in a rapid increase in live coral cover compared with unrestored areas (Williams et al. 2019). Modular metal structures stabilized coral rubble to facilitate natural coral recruitment and structurally support transplanted coral fragments (figure 6). There have been fewer efforts to restore degraded seagrass meadows in Wallacea, although experiments in Spermonde showed that incorporating species diversity into restoration efforts, rather than replanting a single foundation species, had positive effects on ecosystem function and services (Williams et al. 2017). Successful examples of larger-scale seagrass restoration are known from two sites in Java, where meadows of *Enhalus* and *Thalassia* species were replanted and have since persisted for more than a decade (Thorhaug et al. 2020)

Future restoration needs to be implemented at a much larger scale across Wallacea to support the targets of the UN 2021–2030 Decade of Ecosystem Restoration. However, this requires broad consensus across different sectors of society, to help remove barriers such as tenure issues with other land and sea users. Upscaling coral reef and seagrass restoration to the levels required to counteract Indonesia's bomb-fishing legacy and climate change mortality is infeasible, making prevention of ecosystem degradation a better overall strategy than restoration once the damage is done. Indeed, protected natural capital assets will always be better, both ecologically and economically, than restoration. Complementary protocols for long-term monitoring and evaluation of restoration outcomes, including biodiversity, ecosystem functioning, and livelihood benefits, are also needed.

## Conclusions and future research directions

There remains immense potential for emerging scientific and technological tools to further document, monitor, and help prioritize Wallacea's unique and imperiled biodiversity for conservation actions. For example, combining traditional morphological analyses with bioacoustic, genetic, and genomic data led to the description of 12 new bird taxa from Sulawesi's satellite islands, more than double the number typically described per year globally (O'Connell et al. 2019, Rheindt et al. 2020). Molecular methods, including environmental DNA, and large-scale collaborations to catalog the genetic code of thousands of organisms are also facilitating a revolution in biodiversity monitoring. This is particularly notable in coastal ecosystems where microbial surveys in sediment and water help assess their roles in ecosystem functioning (DiBattista et al. 2020) and conservation (Bani et al. 2020). The science of restoration is also being served by population genomic and meta-omic approaches, which can be used to reveal new genotypes and the potential for genetic biocontrol of pest populations as well as for monitoring purposes (Breed et al. 2019). It is now possible to construct highly resolved food webs, moving beyond the study of specific plants or animals, toward a holistic understanding of direct and indirect species interactions that links biodiversity with ecosystem functioning (O'Connell et al. 2022).

As well as taking full advantage of scientific advances, there needs to be a fundamental shift away from managing marine, freshwater, and terrestrial realms independently. This can lead to inefficiencies and inaction, particularly where interactions and threats occur between ecosystems, such as the influence of land-use change on coastal sediment exposure (Beger et al. 2010). Taking a ridge-to-reef approach should lead to lower opportunity costs and improved outcomes for conservation in Wallacea. To be future-proof, land-use and conservation planning should also ensure these ecosystems and the endemic biodiversity they support are climate resilient (Struebig et al. 2015, Dixon et al. 2021).

Scenario planning with multiple, diverse groups of stakeholders, where a range of what-if future land- and marine-use scenarios are explored, can be helpful in identifying and understanding different viewpoints, key knowledge gaps, and the nature of interactions between inputs and outcomes (e.g., do tipping points exist beyond which ecological recovery is unlikely?). Although research can only approximate complex and nonlinear interactions, it can help to avoid inappropriate policy or practice decisions. Other sources of knowledge, such as views on traditional management practices, can also help steer landscape or seascape planning in a direction where people with the greatest role in implementing sustainable practices (i.e., Wallacea's vast and growing rural communities) are given a voice regarding managing their local ecosystems. Indonesia's national planning processes now incorporate scenario tools, and there are moves to expand this to subnational development planning, starting with Sulawesi (Nadin et al. 2019).

Because poverty remains a major driver of unsustainable natural resource use in Wallacea, it should be tackled head on. We need a much better grasp of what evidence and which decisions underpin optimal outcomes for community wellbeing, ensuring that socioeconomic gains from the region's development are not undermined by socioecological losses. For example, the oil palm industry, still in its infancy in Wallacea, has largely helped improve living standards elsewhere in Indonesia but has also resulted in social conflict and pollution in places (Santika et al. 2021). There is huge scope for Indonesia's mining boom to follow the same trajectory if environmental and social safeguards are not adequately followed. Although the government requires all mining permit holders to follow good mining principles and plan for postmining reclamation before they can begin operations, land reclamation is ecologically challenging and mostly oriented toward restoring soils and basic vegetation (Pratiwi et al. 2021). Minimizing the ecological footprint and engaging nearby communities in the first place (e.g., by optimizing land-use planning using the High Conservation Value approach, www.hcvnetwork.org) is clearly a more cost-effective strategy to minimize the impacts of development and maximize the benefits it brings (Budiharta et al. 2018). Improved monitoring and evaluation of mining and postmining operations are needed, to help identify best management practices that can be promoted broadly across the country. After all, Wallacea's central role producing the nickel needed to help the global transition to low carbon technologies should bring investments that benefit local communities, and not the land disputes and environmental damage reported from some sites (Hudayana et al. 2020).

As part of these efforts, community-based and locally led approaches that have been effective elsewhere in Indonesia (Santika et al. 2019) should be further promoted in Wallacea, with communities empowered to derive sustainable livelihoods from these schemes and contribute to conservation. For instance, terrestrial and marine wildlife tourism is already an important source of income for Wallacean communities on Komodo island (Ardiantiono et al. 2018) and in North Sulawesi (Towoliu 2014). In Bangka Island, North Sulawesi, the combined actions of local communities and tourism operators successfully stopped illegal mining activities in favor of sustainable jobs and subsistence fishing (Kalalo 2019). However, tourism is not without challenges as excessive diving also affects reef conditions (Towoliu 2014). Likewise, a key need for Wallacea's terrestrial and marine community-managed areas will be to evaluate sustainable extraction rates and integrate these appropriately in management plans alongside incentives and rewards for sustainable activities. For example, maleo numbers experienced a population recovery in Tompotika, Central Sulawesi, after villages were engaged by local conservationists and received community benefits (financial and nonfinancial) in exchange for ceasing egg poaching (Tasirin et al. 2021).

Although ecosystem restoration licenses appear to be a promising tool, the high costs of restoration have yet to be sufficiently offset via harvestable products, carbon markets, or other payments for ecosystem service schemes (Harrison et al. 2020). The success of ecosystem restoration licenses could be improved if ecosystem services (e.g., carbon, biodiversity, water) were adequately valued and the payments were allocated fairly. Ensuring that payments reach people closest to and most dependent on ecosystem services remains politically challenging. However, Indonesia's government has taken steps in the right direction through its recognition of traditional land and natural resource use rights, and a willingness to transfer management rights from the state to rural communities (Rakatama and Pandit 2020). Clear rights and appropriate rewards may provide people with the security that prevents short-term overexploitation of resources to protect longer-term benefits. Without such changes in thinking, the environmental costs of natural resource management in Wallacea, as it does elsewhere in the world, will ultimately negate most socioeconomic gains.

## Supplementary Material

biac085_Supplemental_File

## Author Biographical

Matthew J. Struebig and Sabhrina G. Aninta contributed equally as joint first author of the manuscript. Matthew J. Struebig (m.j.struebig@kent.ac.uk) is affiliated with the Durrell Institute of Conservation and Ecology, at the University of Kent, in Canterbury, England, in the United Kingdom. Sabhrina G. Aninta (s.g.aninta@qmul.ac.uk) is affiliated with the School of Biological and Behavioural Sciences at Queen Mary University of London, in London, England, in the United Kingdom. Maria Beger (m.beger@leeds.ac.uk) is affiliated with the School of Biology at the University of Leeds, in Leeds, England, in the United Kingdom. Alessia Bani (ab18858@essex.ac.uk) is affiliated with the School of Life Sciences at the University of Essex, in Colchester, England, in the United Kingdom. Henry Barus (henbarus@hotmail.com) is affiliated with the Faculty of Agriculture at Tadulako University, in Palu, Indonesia. Selina Brace (s.brace@nhm.ac.uk) is affiliated with the Department of Earth Sciences at the Natural History Museum, in London, England, in the United Kingdom. Zoe G Davies (z.g.davies@kent.ac.uk) is affiliated with the Durrell Institute of Conservation and Ecology, at the University of Kent, in Canterbury, England, in the United Kingdom. Maarten De Brauwer (maarten.debrauwer@csiro.au) is affiliated with the Commonwealth Scientific and Industrial Research Organisation (CSIRO), Hobart, Australia. Karen Diele (k.diele@napier.ac.uk) is affiliated with the Centre for Conservation and Restoration Science, in the School of Applied Sciences at Edinburgh Napier University, in Edinburgh, Scotland, in the United Kingdom. Cilun Djakiman (cilun.djakimann@gmail.com) is affiliated with the Maritime and Marine Science Center of Excellence at Pattimura University, in Ambon, Indonesia. Rignolda Djamaluddin (rignolda@unsrat.ac.id) is affiliated with the Faculty of Fishery and Marine Science at Sam Ratulangi University, in Manado, Indonesia. Rosie Drinkwater (r.drinkwater@qmul.ac.uk) is affiliated with the School of Biological and Behavioural Sciences, Queen Mary University of London, in London, England, in the United Kingdom. Alex Dumbrell (adumb@essex.ac.uk) is affiliated with the School of Life Sciences at the University of Essex, in Colchester, England, in the United Kingdom. Darren Evans (darren.evans@newcastle.ac.uk) is affiliated with the School of Natural and Environmental Sciences at Newcastle University, in Newcastle upon Tyne, England, in the United Kingdom. Marco Fusi (m.fusi@napier.ac.uk) is affiliated with the Centre for Conservation and Restoration Science, in the School of Applied Sciences at Edinburgh Napier University, in Edinburgh, Scotland, in the United Kingdom. Leonel Herrera-Alsina (leonel.herreraalsina@abdn.ac.uk) is affiliated with the School of Biological Sciences at the University of Aberdeen, in Aberdeen, Scotland, in the United Kingdom. Djoko T. Iskandar (iskandar@sith.itb.ac.id) is affiliated with the School of Life Sciences and Technology at the Bandung Institute of Technology, in Bandung, Indonesia. Jamaluddin Jompa (jamaluddin.jompa@gmail.com) is affiliated with the Faculty of Marine Science and Fisheries at Hasanuddin University in Makassar, Indonesia. Berry Juliandi (bjuliandi@apps.ipb.ac.id) is affiliated with the Department of Biology at Bogor Agricultural Institute, in Bogor, Indonesia. Lesley T. Lancaster (lesleylancaster@abdn.ac.uk) is affiliated with the School of Biological Sciences at the University of Aberdeen, in Aberdeen, Scotland, in the United Kingdom. Gino Limmon (gino.limmon@gmail.com) is affiliated with the Department of Marine Science at Pattimura University, in Ambon, Indonesia. Lindawati (lindawati@surya.ac.id) is affiliated with the Department of Chemical and Green Process Engineering at Surya University, in Tangerang, Indonesia. Michaela G. Y. Lo (mgyl2@kent.ac.uk) is affiliated with the Durrell Institute of Conservation and Ecology, at the University of Kent, in Canterbury, England, in the United Kingdom. Pungki Lupiyaningdyah (pungkilupi@gmail.com) is affiliated with the Research Center for Biosystematics and Evolution at the National Research and Innovation Agency, in Cibinong, Indonesia. Molly McCannon (mollymccannon@hotmail.co.uk) is affiliated with the School of Biology at the University of Leeds, in Leeds, England, in the United Kingdom. Erik Meijaard (e.meijaard@gmail.com) is affiliated with Borneo Futures, in Bandar Seri Begawan, in Brunei Darussalam. Simon L. Mitchell (simonleomitchell@gmail.com) is affiliated with the Durrell Institute of Conservation and Ecology, at the University of Kent, in Canterbury, England, in the United Kingdom. Sonny Mumbunan (mumbunan@gmail.com) is affiliated with the Research Center for Climate Change, University of Indonesia, in Depok, Indonesia. Darren O'Connell (darren.oconnell@ucd.ie) is affiliated with the School of Biology and Environmental Science at University College Dublin, in Dublin, Ireland, Owen G. Osborne (o.osborne@bangor.ac.uk) in the United Kingdom. is affiliated with Molecular Ecology and Evolution Bangor at Bangor University, in Bangor, Wales, in the United Kingdom. Alex S. T. Papadopulos (a.papadopulos@bangor.ac.uk) is affiliated with Molecular Ecology and Evolution Bangor, at Bangor University, in Bangor, Wales, in the United Kingdom. Joeni S. Rahajoe (srjoeni@gmail.com) is affiliated with the Research Center for Ecology and Ethnobiology at the National Research and Innovation Agency, in Cibinong, Indonesia. Rosaria (rosaria.rosaria@ox.ac.id) is affiliated with the Department of Biology at the University of Oxford, in Oxford, England, in the United Kingdom. Stephen J. Rossiter (s.j.rossiter@qmul.ac.uk) is affiliated with the School of Biological and Behavioural Sciences at Queen Mary University of London, in London, England, in the United Kingdom. Rugayah (titikrugayah@yahoo.com) is affiliated with the Herbarium Bogoriense, in the National Research and Innovation Agency, in Cibinong, Indonesia. Himmah Rustiami (hrustiami@gmail.com) is affiliated with the Herbarium Bogoriense, in the National Research and Innovation Agency, in Cibinong, Indonesia. Ulrich Salzmann (ulrich.salzmann@northumbria.ac.uk) is affiliated with the Department of Geography and Environmental Sciences at Northumbria University, in Newcastle upon Tyne, England, in the United Kingdom. Sheherazade (sheherazade.jayadi@gmail.com) is affiliated with the Sulawesi Regional Ecological Conservation Initiative, in Luwuk, Indonesia. I Made Sudiana (imad003@brin.go.jp) is affiliated with the Research Center for Applied Microbiology at the National Research and Innovation Agency, in Cibinong, Indonesia. Endang Sukara (endangsukara@gmail.com) is affiliated with the Graduate School at the University of Indonesia, in Depok, Indonesia. Johny S. Tasirin (jtasirin@unsrat.ac.id) is affiliated with the Department of Forest Sciences at Universitas Sam Ratulangi, in Manado, Indonesia. Aiyen Tjoa (aiyenb@yahoo.com) is affiliated with the Faculty of Agriculture of Tadulako University, in, Palu, Indonesia. Justin M. J. Travis (justin.travis@abdn.ac.uk) is affiliated with the School of Biological Sciences at the University of Aberdeen, in Aberdeen, Scotland, in the United Kingdom. Liam Trethowan (l.trethowan@kew.org) is affiliated with Herbarium Kew, in the Royal Botanic Gardens Kew, in London, England, in the United Kingdom. Agus Trianto (agustrianto.undip@gmail.com) is affiliated with the Faculty of Fisheries and Marine Science at Universitas Diponegoro, Indonesia. Tim Utteridge (t.utteridge@kew.org) is affiliated with Herbarium Kew, in the Royal Botanic Gardens Kew, in London, England, in the United Kingdom. Maria Voigt (m.voigt@kent.ac.uk) is affiliated with the Durrell Institute of Conservation and Ecology, at the University of Kent, in Canterbury, England, in the United Kingdom. Nurul Winarni (nwinarni@gmail.com) is affiliated with the Research Center for Climate Change, at the University of Indonesia, in Depok, Indonesia. Zulianto Zakaria (zuliyanto_zakaria@ung.ac.id) is affiliated with the Department of Biology at Gorontalo University, in Gorontalo City, Indonesia. David P. Edwards (david.edwards@sheffield.ac.uk) is affiliated with the School of Biosciences at the University of Sheffield, in Sheffield, England, in the United Kingdom. Laurent Frantz (laurent.frantz@qmul.ac.uk) is affiliated with the School of Biological and Behavioural Sciences at Queen Mary University of London, in London, England, in the United Kingdom. Jatna Supriatna (jsupriatna@sci.ui.ac.id) is affiliated with the Research Center for Climate Change, at the University of Indonesia, in Depok, Indonesia.
