# Supplementary material for: Safeguarding Imperiled Biodiversity and Evolutionary Processes in the Wallacea Center of Endemism
Source: Bioscience. 2022 Oct 19;72(11):1118–30. doi: 10.1093/biosci/biac085 (PMC9618277; doi:10.1093/biosci/biac085)
Supplement: biac085_Supplemental_File [file biac085_supplemental_file.docx]

***Supplementary Table 1. Characteristics of areas of endemism in Wallacea. Areas are based on the geological information proposed by Michaux (2010), and updated to cover Southeast and Eastern Sulawesi (marked by *).***

| **Island/area** | **Geological process** |  |
| --- | --- | --- |
| **Sulawesi** |  |  |
| a. Northern Sulawesi | Collision of island ~25 Mya; island arc 45 Mya. |  |
| b. South/West Sulawesi | Opening of Makassar strait 45 Mya. |  |
| c. Southeast Sulawesi* | Uplifted and subsided multiple times from ~20-5 Mya |  |
| d. Eastern Sulawesi* | Uplifted and subsided multiple times from ~20-5 Mya |  |
| **Lesser Sundas (*Nusa Tenggara*)** |  |  |
| e. Lombok to Flores/Alor | Mobilism of Sunda taxa onto Sumba block 3-4 Mya; mobilism of Sahul taxa following Timor-Banda collision ~2-4 Mya |  |
| f. Timor, Wetar & Babar | Australian continental margin-Island arc collision ~4-5 Mya; mobilism of taxa ~1-4 Mya |  |
| **Mollucas (*Maluku*)** |  |  |
| g. North: Halmahera | Formation of East Halmahera Arc 45 Mya |  |
| h. Banggai & Sula archipelago | Collision of Sula Spur ~25-10 Mya; continental extension |  |
| i. South-Central Maluku | Fragmentation of Sula Spur <25 Mya |  |

***Supplementary Table 2. Examples of phylogeographic studies of Sulawesi taxa including studies at population, species, and family level. Genetic markers used in these studies include plastid (cpDNA), mitochondrial markers (mtDNA), microsatellites (microSat), nuclear markers (nucDNA), RNA sequencing methods (RNA-seq) and genomic DNA sequencing (gDNA). The references used in the making of figure 3 are marked with *.***

| **Taxon** | **Contact zones** | **Genetic marker** | **Source** |
| --- | --- | --- | --- |
|  |  |  |  |
| **Plants** | | |  |
| *Cyrtandra* | >2 | 4 cpDNA, 1 nucDNA | Atkins et al. (2020) |
| Pitcher plants (*Nepenthes* spp.) | >2 | gDNA | Murphy et al. (2020) |
| **Gastropods** |  |  |  |
| Freshwater snails (*Tylomelania* spp.) | 3 | 2 mtDNA | von Rintelen et al. (2014) |
| **Insects** |  |  |  |
| Crickets (*Chitura* spp.) | 2 | 1 mtDNA | Bridle et al. (2001) |
| Damselflies (Chlorocyphidae) | 4 | Morphological | van Tol (2007) |
| **Fish** |  |  |  |
| *Freshwater fish* | 2 | RNAseq | Montenegro et al. (2019) |
| Ricefish (*Oryzias spp.*) | 4 | 1 mtDNA, gDNA | Mandagi et al (2021) |
| **Amphibians** |  |  |  |
| Celebes toad (*Bufo celebensis*) | 4 | 1 mtDNA, 2 nucDNA | Evans et al. (2008) |
| Fanged frogs (*Limnonectes*) | >2 | 3 mtDNA, 1 nucDNA | Setiadi et al. (2011) |
| **Reptiles** |  |  |  |
| Flying lizards (Draco spp.) | 2 | 2 mtDNA | McGuire et al. (2007)* |
| **Aves** |  |  |  |
| Rusty-bellied Fantail (*Rhipidura teysmanni*) | >3 | 1 mtDNA, gDNA | Ng et al (2017) |
| Sulawesi babblers (*Pellorneum celebense*) | 4 | 2 mtDNA, acoustics | Ó Marcaigh et al. (2021) |
| **Mammals** |  |  |  |
| Anoa (*Bubalus* spp.) | 5 | 2 mtDNA, microSat | Frantz et al. (2018) |
| Fruit bats (Pteropodidae) | >2 | 1 mtDNA***,*** microSat | Campell et al. (2007)* |
| Macaques (*Macaca* spp) | 6 | 1 mtDNA | Evans et al. (2003)* |
| Babirusa (*Babyrousa* spp) | 6 | 2 mtDNA, microSat | Frantz et al. (2018)* |
| Shrews (*Crocidura* spp.) | 2-6 | 1 mtDNA, 15 nucDNA | Eldridge et al. (2018); Esselstyn et al. (2021) |
| Sulawesi warty pig (*Sus celebensis*) | 5 | 2 mtDNA, microSat | Frantz et al (2018) |
| Tarsiers *(Tarsius* spp.) | 4 | 6 nucDNA | Driller et al. (2015)* |

**Supplementary Table S3.** Species used to delineate marine evolutionary breaks and sources in Wallacea. These breaks were then mapped in Figure S1, to delineate the marine regions in Fig 1 of the main manuscript.

| **Taxon** | **Species** | **Source** |
| --- | --- | --- |
| Stomatopod Crustacea (Mantis shrimps)  Actinopterygii (Fishes)  Cardiid Mollusca (Giant clams) | *Haptosquilla pulchella, Haptosquilla glyptocerus, Gonodactylinus viridis*  *Hippocampus trimaculatus, H kuda, Siganus fuscescens, Decapterus russelli, Dascyllus trimaculatus*  *Tridacna crocea, T maxima* | Barber et al. (2011); Carpenter et al. (2011); von der Heyden et al. (2014); Triandiza et al. (2019) |
| Actinopterygii (Fishes) | *Thunnus obesus* | Akbar et al. (2019) |
| Cardiid Mollusca (Giant clams) | *Tridacna crocea, maxima, squamosa* | DeBoer and Barber (2010) |
| Actinopterygii (Fishes) | *Scomberomus commerson* | Jackson et al. (2014) |
| Actinopterygii (Fishes) | *Auxis thazard*  *Sardinella lemuru*  *Rastrelliger kanagurta* | Pedrosa-Gerasmio et al. (2015) |
| Actinopterygii (Fishes) | *Decapterus macarellus* | Zamroni and Suwarso (2018) |
| Scleractinian Cnidaria (Corals) | *Acropora tenuis* | Rosser et al. (2020) |
| Alcyonacean Cnidaria (Soft corals) | *Sarcophyton trocheliophorum* | Kusuma et al. (2016) |
| Scleractinian Cnidaria (Corals) | *Lobophyllia corymbosa* | Umar et al. (2018); Umar et al. (2019) |
| Decapod Crustacea (Crabs) | *Portunus pelagicus* | Hidayani et al. (2020); Madduppa et al. (2021) |
| Cardiid Mollusca (Giant clams) | *Tridacna maxima* | Triandiza et al. (2020) |
| Asteroidea, Echinodermata (Sea stars) | *Linckia laevigata* | Otwoma and Kochzius (2016) |
| Actinopterygii (Fishes) | *Rastrelliger kanaguarta* | Zamroni et al. (2017) |
| Onchidiid Mollusca (Sea slugs) | *Wallaconchis* spp | Goulding et al. (2018) |
| Decapod Arthropoda (Cleaner shrimp) | *Stenopus hispidus* | Wainwright et al. (2020) |
| Actinopterygii (Fishes) | *Eviota sebreei* and sister species | Tornabene et al. (2016) |
| Actinopterygii (Fishes) | *Dascyllus trimaculatus, Amphiprion ocellaris* | Eble et al. (2015) |
| Actinopterygii (Fishes) | *Dascyllus aruanus* | Raynal et al. (2014) |
| Actinopterygii (Fishes) | *Neotrygon kuhlii* | Arlyza et al. (2013) |
| Actinopterygii (Fishes) | *Neotrygon spp group* | Borsa et al. (2016) |
| Actinopterygii (Fishes) | *Plectropomus leopardus* | Ma et al. (2018) |

**Figure S1.** Phylogeographic breaks in Wallacea delineated by blue lines, with the number of lines indicating the number of species found to have breaks, and the colour indicating the taxon. The map depicts an incomplete and conservative pattern of breaks as most studies did not sample comprehensively across the ranges of species examined.


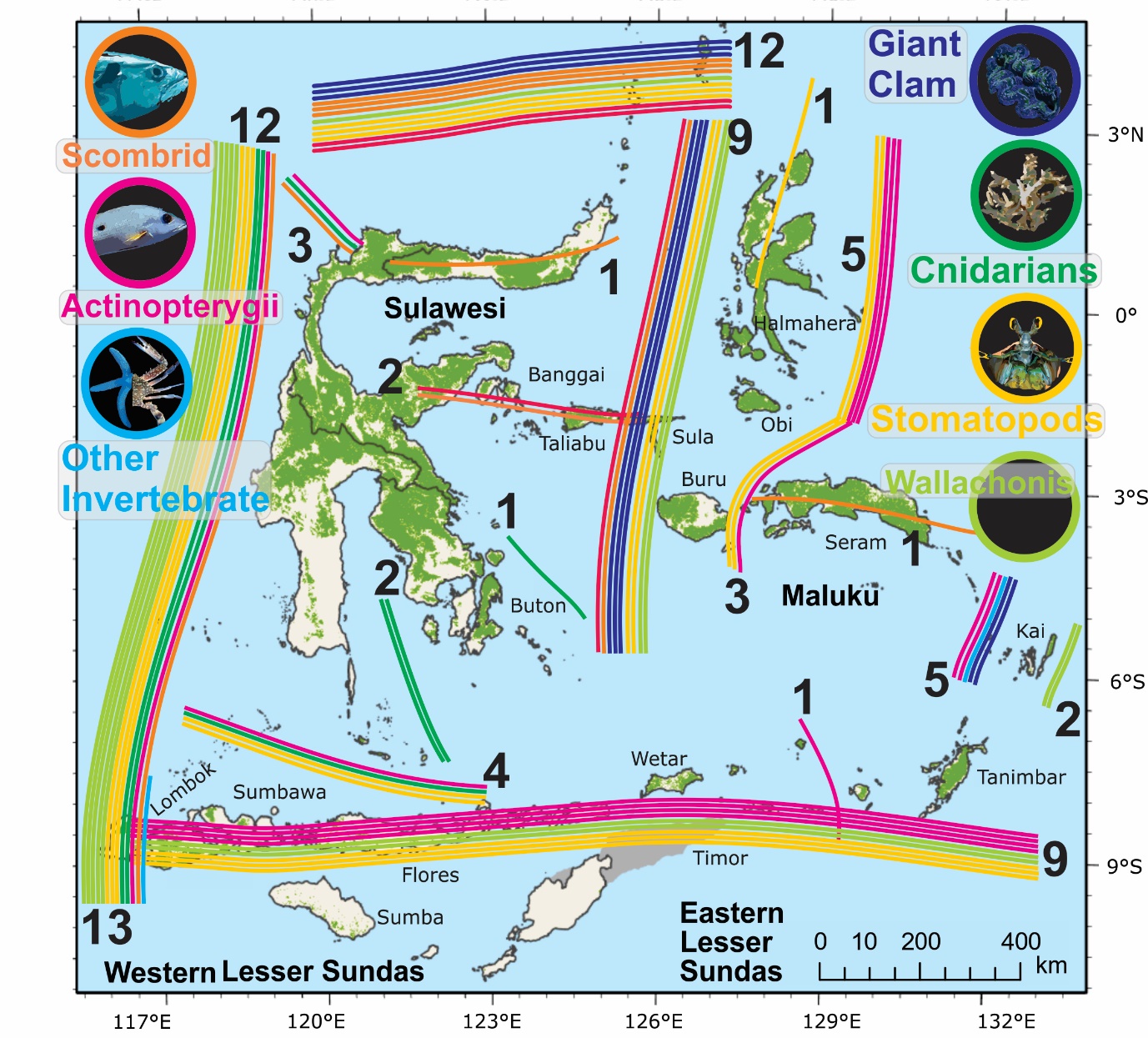


**Supplementary References**

Akbar N, Irfan M, Aris M. 2019. Population Genetics and Phylogeography of Bigeye Tuna in Moluccas Seas, Indonesia. ILMU KELAUTAN: Indonesian Journal of Marine Sciences 23: 145–155.

Arlyza IS, Shen K-N, Durand J-D, Borsa P. 2013. Mitochondrial Haplotypes Indicate Parapatric-like Phylogeographic Structure in Blue-Spotted Maskray (Neotrygon kuhlii) from the Coral Triangle Region. Journal of Heredity 104: 725–733.

Atkins HJ, Bramley GLC, Johnson MA, Kartonegoro A, Nishii K, Kokubugata G, Moeller M, Hughes M. 2020. A molecular phylogeny of Southeast Asian Cyrtandra (Gesneriaceae) supports an emerging paradigm for Malesian plant biogeography. Frontiers of Biogeography 12.

Barber P, Cheng S, Erdmann M, Tenggardjaja K, Ambariyanto. 2011. Evolution and conservation of marine biodiversity in the Coral Triangle: insights from stomatopod Crustacea. Pages 129–156 in Schubart C, ed. Phylogeography and Population Genetics in Crustacea. CRC Press.

Borsa P, Shen K-N, Arlyza IS, Hoareau TB. 2016. Multiple cryptic species in the blue-spotted maskray (Myliobatoidei: Dasyatidae: Neotrygon spp.): An update. Comptes Rendus Biologies 339: 417–426.

Bridle JR, Garn A-K, Monk KA, Butlin RK. 2001. Speciation in Chitaura grasshoppers (Acrididae: Oxyinae) on the island of Sulawesi: colour patterns, morphology and contact zones. Biological Journal of the Linnean Society 72: 373–390.

Campbell, P., Putnam, A. S., Bonney, C., Bilgin, R., Morales, J. C., Kunz, T. H., & Ruedas, L. A. (2007). Contrasting patterns of genetic differentiation between endemic and widespread species of fruit bats (Chiroptera: Pteropodidae) in Sulawesi, Indonesia. Molecular Phylogenetics and Evolution, *44*: 474-482.

Carpenter KE, Barber PH, Crandall ED, Ablan-Lagman MCA, Ambariyanto, Mahardika GN, Manjaji-Matsumoto BM, Juinio-Meñez MA, Santos MD, Starger CJ, Toha AHA. 2011. Comparative Phylogeography of the Coral Triangle and Implications for Marine Management. Journal of Marine Biology 2011: e396982.

DeBoer TS, Barber PH. 2010. Isolation and characterization of 9 polymorphic microsatellite markers for the endangered boring giant clam (Tridacna crocea) and cross-priming testing in three other Tridacnid species. Conservation Genetics Resources 2: 353–356.

Driller C, Merker S, Perwitasari-Farajallah D, Sinaga W, Anggraeni N, Zischler H. 2015. Stop and Go – Waves of Tarsier Dispersal Mirror the Genesis of Sulawesi Island. PLOS ONE 10: e0141212.

Eble JA, Bowen BW, Bernardi G. 2015. Phylogeography of coral reef fishes. Pages 64–75 in Mora C, ed. Ecology of Fishes on Coral Reefs. Cambridge University Press.

Eldridge RA, Achmadi AS, Giarla TC, Rowe KC, Esselstyn JA. 2018. Geographic isolation and elevational gradients promote diversification in an endemic shrew on Sulawesi. Molecular Phylogenetics and Evolution 118: 306–317.

Esselstyn JA, Achmadi AS, Handika H, Swanson MT, Giarla TC, Rowe KC. 2021. Fourteen New, Endemic Species of Shrew (Genus Crocidura) from Sulawesi Reveal a Spectacular Island Radiation. Bulletin of the American Museum of Natural History 454.

Evans BJ, McGuire JA, Brown RM, Andayani N, Supriatna J. 2008. A coalescent framework for comparing alternative models of population structure with genetic data: evolution of Celebes toads. Biology Letters 4: 430–433.

Evans BJ, Supriatna J, Andayani N, Setiadi MI, Cannatella DC, Melnick DJ. 2003. Monkeys and Toads Define Areas of Endemism on Sulawesi. Evolution 57: 1436–1443.

Fortuin AR, Van der Werff W., Wensink H. 1997. Neogene basin history and paleomagnetism of a rifted and inverted forearc region, on- and offshore Sumba, Eastern Indonesia. Journal of Asian Earth Sciences 15: 61–88.

Frantz, L. A., Rudzinski, A., Nugraha, A. M. S., Evin, A., Burton, J., Hulme-Beaman, A., ... & Larson, G. (2018). Synchronous diversification of Sulawesi's iconic artiodactyls driven by recent geological events. Proceedings of the Royal Society B: Biological Sciences, 285: 20172566.

Goulding TC, Khalil M, Tan SH, Dayrat B. 2018. Integrative taxonomy of a new and highly-diverse genus of onchidiid slugs from the Coral Triangle (Gastropoda, Pulmonata, Onchidiidae). ZooKeys 763: 1–111.

von der Heyden S, Beger M, Toonen R, Herwerden L, Juinio-Meñez M, Ravago-Gotanco R, Fauvelot C, Bernardi G. 2014. The application of genetics to marine management and conservation: Examples from the Indo-Pacific. Bulletin of Marine Science 90.

Hidayani AA, Fujaya Y, Trijuno DD, Rukminasari N, Alimuddin A. 2020. Genetic diversity of blue swimming crab (Portunus pelagicus Linn 1758) from Indonesian waters (Sunda and Sahul Shelf, Wallacea region): Phylogenetic approach. Biodiversitas Journal of Biological Diversity 21.

Jackson AM, Ambariyanto, Erdmann MV, Toha AHA, Stevens LA, Barber PH. 2014. Phylogeography of commercial tuna and mackerel in the Indonesian Archipelago. Bulletin of Marine Science 90: 471–492.

Kaneko Y, Maruyama S, Kadurasman A, Ota T, Ishikawa M, Tsujimori T, Ishikawa A, Okamoto K. 2007. Ongoing orogeny in the outer-arc of the Timor–Tanimbar region, eastern Indonesia. Gondwana Research. 11: 218–233.

Kusuma AB, Bengen DG, Madduppa H, Subhan B, Arafat D, Negara BFSP. 2016. Close genetic connectivity of soft coral Sarcophyton trocheliophorum in Indonesia and its implication for marine protected area. Aceh Journal of Animal Science 1: 50–57.

Ma KY, van Herwerden L, Newman SJ, Berumen ML, Choat JH, Chu KH, Sadovy de Mitcheson Y. 2018. Contrasting population genetic structure in three aggregating groupers (Percoidei: Epinephelidae) in the Indo-West Pacific: the importance of reproductive mode. BMC Evolutionary Biology 18: 180.

Madduppa H, Martaulina R, Zairion Z, Renjani RM, Kawaroe M, Anggraini NP, Subhan B, Verawati I, Sani LMI. 2021. Genetic population subdivision of the blue swimming crab (Portunus pelagicus) across Indonesia inferred from mitochondrial DNA: Implication to sustainable fishery. PLOS ONE 16: e0240951.

Mandagi IF, Kakioka R, Montenegro J, Kobayashi H, Masengi KW, Inomata N, Yamahira, K. (2021). Species divergence and repeated ancient hybridization in a Sulawesian lake system. Journal of Evolutionary Biology, 34: 1767-1780.

McGuire JA, Brown, RM, Riyanto, A, Andayani N. 2007. The flying lizards of the Draco lineatus group (Squamata: Iguania: Agamidae): a taxonomic revision with descriptions of two new species. Herpetological Monographs, 21: 179-212.

Michaux B. 2010. Biogeology of Wallacea: geotectonic models, areas of endemism, and natural biogeographical units. Biological Journal of the Linnean Society 101: 193–212.

Montenegro J, Mochida K, Matsui K, Mokodongan DF, Sumarto BKA, Lawelle SA, Nofrianto AB, Hadiaty RK, Masengi KWA, Yong L, Inomata N, Irie T, Hashiguchi Y, Terai Y, Kitano J, Yamahira K. 2019. Convergent evolution of body color between sympatric freshwater fishes via different visual sensory evolution. Ecology and Evolution 9: 6389–6398.

Murphy B, Forest F, Barraclough T, Rosindell J, Bellot S, Cowan R, Golos M, Jebb M, Cheek M. 2020. A phylogenomic analysis of Nepenthes (Nepenthaceae). Molecular Phylogenetics and Evolution 144: 106668.

Ng, N. S., Wilton, P. R., Prawiradilaga, D. M., Tay, Y. C., Indrawan, M., Garg, K. M., & Rheindt, F. E. (2017). The effects of Pleistocene climate change on biotic differentiation in a montane songbird clade from Wallacea. Molecular Phylogenetics and Evolution, 114: 353-366

Nguyen N, Duffy B, Shulmeister J, Quigley M. 2013. Rapid Pliocene uplift of Timor. Geology: 179–182.

Otwoma LM, Kochzius M. 2016. Genetic Population Structure of the Coral Reef Sea Star Linckia laevigata in the Western Indian Ocean and Indo-West Pacific. PLOS ONE 11: e0165552.

Ó Marcaigh F, Kelly DJ, O’Connell DP, Dunleavy D, Clark A, Lawless N, Karya A, Analuddin K, Marples NM. 2021. Evolution in the understorey: The Sulawesi babbler Pellorneum celebense (Passeriformes: Pellorneidae) has diverged rapidly on land-bridge islands in the Wallacean biodiversity hotspot. Zoologischer Anzeiger 293: 314–325.

Pedrosa-Gerasmio IR, Agmata AB, Santos MD. 2015. Genetic diversity, population genetic structure, and demographic history of Auxis thazard (Perciformes), Selar crumenophthalmus (Perciformes), Rastrelliger kanagurta (Perciformes) and Sardinella lemuru (Clupeiformes) in Sulu-Celebes Sea inferred by mitochondrial DNA sequences. Fisheries Research 162: 64–74.

Raynal J, Crandall E, Barber P, Mahardika G, Lagman M, Carpenter K. 2014. Basin isolation and oceanographic features influencing lineage divergence in the humbug damselfish <I>(Dascyllus aruanus)</I> in the Coral Triangle. Bulletin of Marine Science 90: 513–532.

von Rintelen T, Stelbrink B, Marwoto RM, Glaubrecht M. 2014. A Snail Perspective on the Biogeography of Sulawesi, Indonesia: Origin and Intra-Island Dispersal of the Viviparous Freshwater Gastropod Tylomelania. PLOS ONE 9: e98917.

Rosser NL, Edyvane K, Malina AC, Underwood JN, Johnson MS. 2020. Geography and spawning season drive genetic divergence among populations of the hard coral Acropora tenuis from Indonesia and Western Australia. Coral Reefs 39: 989–999.

Setiadi MI, McGuire JA, Brown RM, Zubairi M, Iskandar DT, Andayani N, Supriatna J, Evans BJ. 2011. Adaptive Radiation and Ecological Opportunity in Sulawesi and Philippine Fanged Frog (Limnonectes) Communities. The American Naturalist 178: 221–240.

van Tol J. 2007. The Odonata of Sulawesi and adjacent islands. Part 7. *Libellago* and *Sclerocypha* (Chlorocyphidae). International Journal of Odonatology 10: 209–248.

Tornabene L, Valdez S, Erdmann MV, Pezold FL. 2016. Multi-locus sequence data reveal a new species of coral reef goby (Teleostei: Gobiidae: Eviota), and evidence of Pliocene vicariance across the Coral Triangle. Journal of Fish Biology 88: 1811–1834.

Triandiza T, Kusnadi A, Sari N, Pesilette RN. 2020. Keragaman genetik kima kecil (Tridacna maxima) di Pulau Kur, Pulau Biak, dan Manado serta implikasinya untuk konservasi. Jurnal Penelitian Perikanan Indonesia 26: 167–179.

Triandiza T, Zamani NP, Madduppa H, Hernawan UE. 2019. Distribution and abundance of the giant clams (Cardiidae: Bivalvia) on Kei Islands, Maluku, Indonesia. Biodiversitas Journal of Biological Diversity 20: 884–892.

Umar W, Jompa J, Tassakka ACMAR. 2018. Genetic Diversity and Geographical Gene Flow Patterns of Spawning Broadcast Coral Lobophyllia corymbosa in The Sulawesi Waters as A Coral Triangle Area. IOP Conference Series: Earth and Environmental Science 116: 012060.

Umar W, Tassakka ACM a. R, Jompa J. 2019. High genetic connectivity in a scleractinian coral (Lobophyllia corymbosa) around Sulawesi, Indonesia. Biodiversitas Journal of Biological Diversity 20.

Wainwright BJ, Arlyza IS, Karl SA. 2020. Population genetics of the banded coral shrimp, Stenopus hispidus (Olivier, 1811), in the Indonesian archipelago. Journal of Experimental Marine Biology and Ecology 525: 151325.

Zamroni A, Suwarso S. 2018. Genetic structure of mackerel scad populations (Decapterus macarellus Cuvier, 1833) around Sulawesi based on mtDNA marker, Indonesian Fisheries Research Journal 23: 89.

Zamroni A, Suwarso S, Kuswoyo A. 2017. Variasi genetika ikan banyar, Rastrelliger kanagurta (Cuvier, 1817) di perairan Indonesia Timur. BAWAL Widya Riset Perikanan Tangkap 9: 123–131.
